# Support for Over-the-Counter HIV Preexposure Prophylaxis Among Transfeminine People

**DOI:** 10.1001/jamanetworkopen.2025.27800

**Published:** 2025-08-20

**Authors:** Lauren R. Violette, Samuel M. Jenness, Travis H. Sanchez, Douglas S. Krakower, Julia L. Marcus

**Affiliations:** 1 Department of Population Medicine, Harvard Medical School, Boston, Massachusetts; 2 Department of Epidemiology, Rollins School of Public Health, Emory University, Atlanta, Georgia

## Abstract

This survey study assesses whether transfeminine people would support availability of over-the-counter HIV preexposure prophylaxis.

## Introduction

As policy threats to health escalate in the US, strategies are urgently needed to sustain access to evidence-based care, particularly for marginalized populations. Transgender populations are disproportionately impacted by HIV, but current delivery models for preexposure prophylaxis (PrEP)^1^ have resulted in barriers to use, including stigma and discrimination, frequent clinical visits and laboratory testing, and cost and insurance-related challenges.^2^ In settings where health systems underserve vulnerable populations, the World Health Organization recommends self-care interventions, such as over-the-counter (OTC) oral contraceptives and HIV self-testing.^3^ If approved, OTC availability of safe, low-cost PrEP could facilitate self-care for HIV prevention by eliminating the burden and costs of clinical visits and laboratory-based testing.^4^ However, whether populations likely to benefit would support this approach is unknown.

## Methods

This survey study analyzed data from the 2023-2024 Transgender Women’s internet Survey and Testing study, an online cross-sectional survey of transgender women and transfeminine nonbinary people (eMethods in Supplement 1).^5^ The Emory University institutional review board approved this study. Informed consent was obtained electronically before survey completion. No incentive was provided to respondents. The AAPOR reporting guideline was followed.

Among those who had never had a positive HIV test result, we asked, “If research showed that using daily oral PrEP without a prescription was safe and effective, would you support making daily oral PrEP available at a pharmacy or online as an over-the-counter product?” We used modified Poisson regression with robust SEs to compute unadjusted prevalence ratios (PRs) with 95% CIs to identify correlates of support for OTC PrEP among all respondents and to evaluate associations between reasons for nonuse of PrEP and support for OTC PrEP among PrEP-naive respondents. SAS, version 9.4 was used for analysis.

## Results

Of 1936 respondents, 1684 (87.0%) were PrEP naive. Overall, 1677 (86.6%) supported OTC PrEP availability, 127 (6.6%) did not support it, 114 (5.9%) were unsure, and 18 (0.9%) preferred not to answer.

Support for OTC PrEP was more prevalent among respondents who had ever taken PrEP (PR, 1.04; 95% CI, 1.01-1.07) or tested for HIV (PR, 1.04; 95% CI, 1.01-1.07) (Table 1). All respondents recently diagnosed with a bacterial sexually transmitted infection supported OTC PrEP. Compared with respondents reporting 0 to 1 sex partners in the prior 12 months, support was higher among those reporting 2 to 9 (PR, 1.04; 95% CI, 1.01-1.07) or more than 10 (PR, 1.09; 95% CI, 1.05-1.12) partners. Support was greater among respondents who had ever worried that a health care practitioner would treat them differently due to the respondent’s gender identity (PR, 1.04; 95% CI, 1.01-1.07) or sexual partnerships (PR, 1.03; 95% CI, 1.01-1.06).

**Table 1.** Factors Associated With Support for OTC PrEP Among Respondents in the 2023-2024 Transgender Women’s Internet Survey and Testing Study

Characteristic	Respondents		Unadjusted PR (95% CI)^b^
	Total, No. (%) (N = 1936)^a^	Supporting OTC PrEP, No./total No. (%) (n = 1677)	
Age, y			
15-24	971 (50.2)	834/900 (92.7)	0.98 (0.93-1.02)
25-29	429 (22.2)	375/408 (91.9)	0.97 (0.93-1.02)
30-39	377 (19.5)	328/348 (94.2)	1.00 (0.95-1.04)
≥40	159 (8.2)	140/148 (94.6)	1 [Reference]
Race and ethnicity^c^			
American Indian or Alaska Native	10 (0.5)	5/8 (62.5)	0.67 (0.39-1.14)
Asian	47 (2.4)	39/44 (88.6)	0.95 (0.85-1.06)
Black or African American	17 (0.9)	15/17 (88.2)	0.94 (0.79-1.12)
Hispanic, Latino, or Spanish^d^	78 (4.0)	68/75 (90.7)	0.97 (0.90-1.05)
White	1447 (74.7)	1260/1348 (93.5)	1 [Reference]
Multiracial	307 (15.9)	265/282 (94.0)	1.01 (0.97-1.04)
Other^e^	7 (0.4)	5/7 (71.4)	0.76 (0.48-1.22)
Health insurance			
Yes	1781 (92.0)	1546/1661 (93.1)	1 [Reference]
No	155 (8.0)	131/143 (91.6)	0.98 (0.94-1.04)
Ever taken PrEP			
Yes	219 (11.3)	196/203 (96.6)	1.04 (1.01-1.07)
No	1684 (87.0)	1459/1577 (92.5)	1 [Reference]
Ever tested for HIV			
Yes	1104 (57.0)	979/1039 (94.2)	1.04 (1.01-1.07)
No	769 (39.7)	647/713 (90.7)	1 [Reference]
Sex partners in past 12 mo among those who reported sex, No.^f^			
0-1	828/1642 (50.4)	704/772 (91.2)	1 [Reference]
2-9	714/1642 (43.5)	627/663 (94.6)	1.04 (1.01-1.07)
≥10	100/1642 (6.1)	95/96 (99.0)	1.09 (1.05-1.12)
Diagnosed with bacterial STI in past 12 mo			
Yes	44 (2.3)	38/38 (100)	1.08 (1.06-1.09)
No	1892 (97.7)	1639/1766 (92.8)	1 [Reference]
Ever worried that a health care practitioner would treat them differently if they told them about their gender identity			
Yes	1437 (74.2)	1255/1338 (93.8)	1.04 (1.00-1.07)
No	466 (24.1)	393/435 (90.3)	1 [Reference]
Ever worried that a health care practitioner would treat them differently if they told them about who they have sex with			
Yes	705 (36.4)	619/653 (94.8)	1.03 (1.01-1.06)
No	1182 (61.0)	1015/1105 (91.9)	1 [Reference]

Abbreviations: OTC, over-the-counter; PR, prevalence ratio; PrEP, preexposure prophylaxis; STI, sexually transmitted infection.

^a^ Numbers might not sum to column total because of missing data.

^b^ Multivariable analyses to adjust for confounding were not conducted because causal relationships were not assessed in this cross-sectional analysis.

^c^ Race and ethnicity were self-reported and ascertained in the same question. These variables were included in the analysis to allow assessment of differences in support for OTC PrEP across subgroups.

^d^ Respondents who only identified as Hispanic, Latino, or Spanish were classified as such. Respondents who identified as Hispanic, Latino, or Spanish and another race were classified as multiracial.

^e^ Includes Armenian, Irish, Jewish, Middle Eastern or North African, and Romani.

^f^ Includes oral, anal, and/or vaginal sex.

Among 1577 PrEP-naive respondents, support for OTC PrEP was greater among those who had never taken PrEP because they did not know enough about it (PR, 1.04; 95% CI, 1.00-1.07), did not know which clinician to see about starting PrEP (PR, 1.06; 95% CI, 1.03-1.09), or were worried about privacy while on a parent’s health plan (PR, 1.06; 95% CI, 1.03-1.09) (Table 2). Respondents who reported PrEP-related stigma from health care practitioners (PR, 1.05; 95% CI, 1.01-1.09) or others (PR, 1.07; 95% CI, 1.04-1.10) indicated greater support.

**Table 2.** Reasons for Never Having Used PrEP and Associations With Support for OTC PrEP Among PrEP-Naive Respondents in the 2023-2024 Transgender Women’s Internet Survey and Testing Study^a^

Reason	Respondents supporting OTC PrEP, No./total No. (%) (n = 1459)	Unadjusted PR (95% CI)^b^
Not sexually active	417/452 (92.3)	1.00 (0.97-1.03)
Monogamous relationship with HIV-negative partner	671/738 (90.9)	0.97 (0.94-0.99)
Too expensive	135/142 (95.1)	1.03 (0.99-1.07)
Do not have health insurance	84/88 (95.4)	1.03 (0.99-1.08)
Do not have reliable transportation	67/75 (89.3)	0.96 (0.89-1.04)
Do not know enough about PrEP to decide	323/340 (95.0)	1.04 (1.00-1.07)
Do not know which clinician to see about starting PrEP	216/222 (97.3)	1.06 (1.03-1.09)
Worried clinician will treat them poorly if they ask about PrEP	96/99 (97.0)	1.05 (1.01-1.09)
Concerned about adverse effects	152/159 (95.6)	1.04 (1.00-1.08)
Cannot remember to take it	106/108 (98.1)	1.07 (1.03-1.10)
Prefer other prevention methods	198/209 (94.7)	1.03 (0.99-1.07)
Worried people will think they have HIV	54/56 (96.4)	1.04 (0.99-1.10)
Worried people will think they are very sexually active	70/71 (98.6)	1.07 (1.04-1.10)
Worried about privacy with someone they live with	97/101 (96.0)	1.04 (1.00-1.09)
Worried about privacy on parent’s health plan	126/129 (97.7)	1.06 (1.03-1.09)

Abbreviations: OTC, over-the-counter; PR, prevalence ratio; PrEP, preexposure prophylaxis.

^a^ Omits 107 PrEP-naive respondents who were unsure or preferred not to answer the question about support for OTC PrEP.

^b^ Reference groups for PRs were respondents who did not endorse each respective reason. Multivariable analyses to adjust for confounding were not conducted because causal relationships were not assessed in this cross-sectional analysis.

## Discussion

Given efforts to erase transgender health from the national research agenda, restrict gender-affirming care, and dismantle discrimination protections for gender-diverse populations,^6^ self-care strategies that can withstand such policy changes are needed. Our results suggest that OTC PrEP could benefit transfeminine people by expanding access to HIV prevention beyond clinic-based settings.

Although this is one of the largest national samples of transfeminine people, with respondents from all 50 states, most respondents were young, White, and insured, limiting generalizability. Further research is needed on OTC PrEP, including interest in use, ability and willingness to pay, and the extent to which OTC PrEP can be used safely and effectively, among transfeminine people and other populations likely to benefit.

## Notes

Supplement 1. **eMethods.** Description of the Transgender Women’s Internet Survey and Testing Study

Supplement 2.
Data Sharing Statement
