# Supplementary material for: Support for Over-the-Counter HIV Preexposure Prophylaxis Among Transfeminine People
Source: JAMA Netw Open. 2025 Aug 20;8(8):e2527800. doi: 10.1001/jamanetworkopen.2025.27800 (PMC12368689; doi:10.1001/jamanetworkopen.2025.27800)
Supplement: Supplement 1. — eMethods. Description of the Transgender Women’s Internet Survey and Testing Study [file jamanetwopen-e2527800-s001.pdf]

Supplemental Online Content

Violette LR, Jenness SM, Sanchez TH, Krakower DS, Marcus JL. Support for over-the-counter HIV preexposure prophylaxis among transfeminine people. *JAMA Netw Open.* 2025;8(8):e2527800. doi:10.1001/jamanetworkopen.2025.27800

eMethods. Description of the Transgender Women's Internet Survey and Testing Study

This supplemental material has been provided by the authors to give readers additional information about their work.

© 2025 Violette LR et al. *JAMA Netw Open*.

eMethods. Description of the Transgender Women's Internet Survey and Testing Study

The Transgender Women's Internet Survey and Testing (TWIST) study is an annual, cross-sectional, online behavioral survey of transgender women and transfeminine non-binary people assigned male at birth who are ≥15 years old, report ever having sex, and reside in the United States. Respondents were recruited through online convenience sampling using banner advertisements on dating applications (e.g., Grindr) and social media sites (e.g., Instagram) from December 2023 through May 2024. The survey collected data on self-reported sociodemographic characteristics, sexual health, mental health, access to care, and stigma. Respondents can opt into receiving mail-in self-testing kits for HIV, sexually transmitted infections, hepatitis C, and hormone levels. Respondents provide informed consent electronically before survey completion. All study procedures and materials were approved by the Emory University Institutional Review Board. Results reporting adhered to American Association for Public Opinion Research guidelines.

© 2025 Violette LR et al. *JAMA Netw Open*.
