# Supplementary material for: Support for Over-the-Counter HIV Preexposure Prophylaxis Among Transfeminine People
Source: JAMA Netw Open. 2025 Aug 20;8(8):e2527800. doi: 10.1001/jamanetworkopen.2025.27800 (PMC12368689; doi:10.1001/jamanetworkopen.2025.27800)
Supplement: Supplement 2. — Data Sharing Statement [file jamanetwopen-e2527800-s002.pdf]

Data Sharing Statement

Violette. Support for Over-the-Counter HIV Preexposure Prophylaxis Among Transfeminine People. *JAMA Netw Open*. Published August 20, 2025.
doi:10.1001/jamanetworkopen.2025.27800

Data
Data available: Yes
Data types: Deidentified participant data
How to access data: travis.sanchez@emory.edu
When available: With publication

Supporting Documents
Document types: None

Additional Information
Who can access the data: researchers whose proposed use of the data has been approved
Types of analyses: for a specified purpose
Mechanisms of data availability: after approval of a proposal
